# Supplementary material for: Amaryllidaceae-Type Alkaloids from Pancratium maritimum: Apoptosis-Inducing Effect and Cell Cycle Arrest on Triple-Negative Breast Cancer Cells
Source: Molecules. 2022 Sep 6;27(18):5759. doi: 10.3390/molecules27185759 (PMC9501014; doi:10.3390/molecules27185759)
Supplement: Supplementary file 1 [file molecules-27-05759-s001.zip › molecules-1862240-supplementary.pdf]

Supplementary Information

Amaryllidaceae-type alkaloids from *Pancratium maritimum*: apoptosis-inducing effect and cell cycle arrest on triple-negative breast cancer cells

Shirley A.R. Sancha[1], Adriana Gomes[2], Joana B. Loureiro[2], Lucília Saraiva[2*], Maria-José U. Ferreira[1*].

[1]Research Institute for Medicines (iMed.ULisboa), Faculty of Pharmacy, University of Lisbon, Av. Prof. Gama Pinto, 1649-003 Lisbon, Portugal.

[2] LAQV/REQUIMTE, Department of Biological Sciences, Laboratory of Microbiology, Faculty of Pharmacy, University of Porto, Rua de Jorge Viterbo Ferreira 228, Porto, 4050-313, Portugal

* Corresponding authors: e-mail address: mjuferreira@ff.ulisboa.pt (Maria-José U. Ferreira); lucilia.saraiva@ff.up.pt

Table of contents

Figure S1: Extraction and fractionation of methanol extract of *P. maritimum*, and the isolated compounds (**1-11**). S2

Table S1: Fractions obtained from acid-base extraction........ S3

Spectroscopic data of compounds S3

NMR spectra of some compounds S7

References........ S16

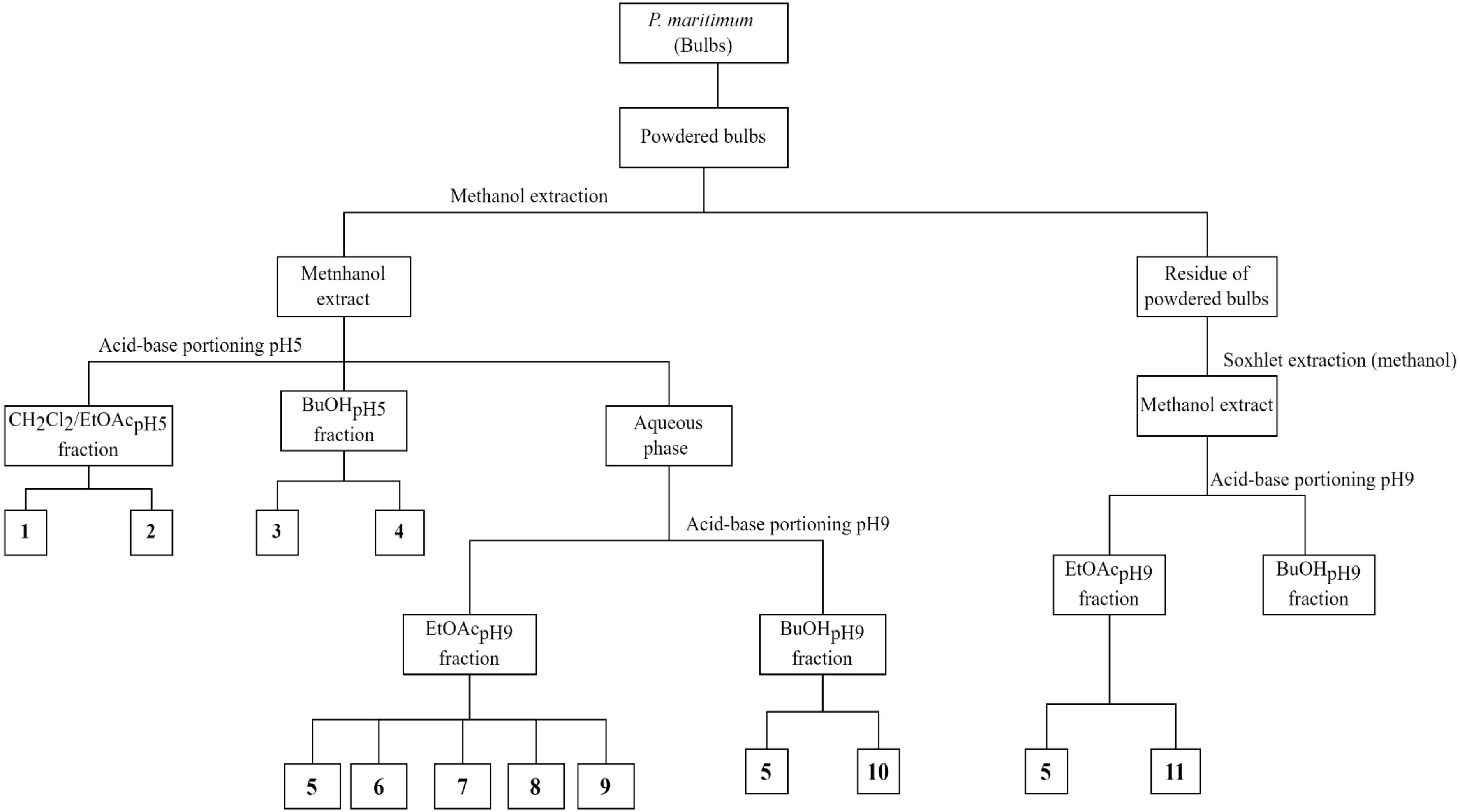

Figure S1: Extraction and fractionation of methanol extract of *P. maritimum,* and the isolated compounds (**1-11**).

Table S1: Fractions obtained from acid-base extraction.

	Quantity (g)	
**Fractions**	Bulbs	Soxhlet extraction
CH_2Cl_{2pH5}	13.9	---
$EtOAc_{pH5}$	20	---
n-$BuOH_{pH5}$	109	---
$EtOAc_{pH9}$	10.3	3.5
n-$BuOH_{pH9}$	32.2	4.5

Spectroscopic data of compounds

4,6-dimethoxy-2-hydroxy-acetophenone (1)

Amorphous powder. ESI-MS (positive mode) m/z (rel.int) 197 $[M + H]^+$ (100). ^1H NMR (300 MHz, $CDCl_3$) δ 14.0 (*s*, 2-OH), 6.05 (1H, *d*, *J* = 2.4 Hz, H-3), 5.91 (*d*, *J* = 2.4 Hz, H-5), 3.85 (*s*, OCH_3), 3.81 (*s*, OCH_3), 2.60 (*s*, CO$\underline{CH_3}$), ppm. ^{13}C NMR (75 MHz, $CDCl_3$) δ 203.2 ($\underline{CO}CH_3$), 167.7 (C-2), 166.2 (C-4), 163.0 (C-6), 106.1 (C-1), 93.6 (C-3), 90.9 (C-5), 55.7 (6-OCH_3), 55.5 (6-OCH_3), 33.0 (CO$\underline{CH_3}$), ppm.

***N-trans*-feruloyl-tyramine (2)**

White crystals, mp: 90-93 ºC (hexane/EtOAc). (lit. 91 ºC) [1]. IR, $\nu_{max,}$ cm^{-1} (KBr): 3495, 3363, 3229, 1647, 1548 cm^{-1}. ESI-MS (positive mode) m/z (rel. int) 330 $[M + H]^+$ (100). ^1H NMR (300 MHz, $CDCl_3$/ CD_3OD; 4:1) δ 7.48 (1H, *d*, *J* = 15.6 Hz, H-3), 7.05 (2H, *d*, *J* = 8.6 Hz H-4′/H-8′), 7.02 (2H, *d*, *J* = 1.7 Hz H-5), 7.01 (1H, *dd*, *J* = 8.5, 1.7 Hz H-9), 6.84 (2H, *d*, *J* = 8.5 Hz, H-5′/ H-7′), 6.78 (1H, *d*, *J* = 8.3 Hz, H-8), 6.29 (1H, *d*, *J* = 15.6 Hz, H-2), 3.88 (3H, *s*, O$\underline{CH_3}$), 3.53 (2H, *t*, *J* = 7.1 Hz, H-1′), 2.78 (2H, *t*, *J* = 7.1 Hz, H-2′), ppm. ^{13}C NMR (75 MHz, $CDCl_3$/ CD_3OD, 4:1) δ 167.6 (C-1), 155.5 (C6′), 148.1 (C-6), 147.6 (C-7), 141.3 (C-3), 130.0 (C-3′′), 129.9 (C-4′/C-8′), 127.1 (C-4), 122.3 (C-9), 117.7 (C-2), 115.5 (C-5′), 115.3 (C-7′), 110.4 (C-5/C-8), 55.8 (O$\underline{CH_3}$), 41.3 (C-1′′), 34.7 (C-2′), ppm.

Haemanthidine (3)

Amorphous powder. $[\alpha]_D^{25}$ − 58.9 (*c* 0.28, $CHCl_3$); lit. $[\alpha]_D^{25}$ − 41 ($CHCl_3$) [2]. ESI-MS (positive mode) m/z (rel. int) 318 $[M + H]^+$ (100). ^1H NMR (300 MHz, $CDCl_3$/CD_3OD, 4:1; epimer A, β-OH) δ 6.86 (1H, *s*, H-7), 6.79 (1H, *s*, H-10), 6.45 (1H, *d*, *J* = 5.4 Hz, H-1), 6.28

(1H, *dd*, *J* = 5.1, 1.1 Hz, H-2) 5.92 (2H, *m*, OCH_2O), 4.93 (1H, *s*, H-6), 3.93 (3H, *m*, H-3/H-11), 3.66 (1H, *dd*, *J* = 13.5, 4.7 Hz, H-4a), 3.37 (3H, *s*, OCH_3), 3.31 (1H, *dd*, *J* = 14.2, 7.4 Hz, H-12-exo), 3.13 (*dd*, *J* = 14.2, 3.2 Hz, H-12-endo), 2.31 (1H, *td*, *J* = 13.6, 4.4 Hz, H-4α), 1.98 (1H, *m*, H-4β), ppm.^{1}H NMR (300 MHz, $CDCl_3/CD_3OD$, 4:1; epimer B, α-OH) δ 6.91 (1H, *s*, H-7), 6.83 (1H, *s*, H-10), 6.42 (1H, *d*, *J* = 5.4 Hz, H-1), 6.24 (1H, *dd*, *J* = 5.1, 1.0 Hz, H-2), 5.92 (2H, *m*, OCH_2O), 5.51 (1H, *s*, H-6), 4.07 (1H, *dd*, *J* = 14.2, 6.8 Hz, H-12-exo), 3.93 (3H, *m*, H-3/H-11), 3.46 (*dd*, *J* = 13.2, 5.0 Hz, H-4a), 3.38 (3H, *s*, OCH_3), 2.82 (1H, *dd*, *J* = 14.3, 2.6 Hz, H-12-endo), 2.17 (1H, *dd*, *J* = 13.7, 4.4 Hz, H-4α), 1.98 (1H, *m*, H-4β), ppm. ^{13}C NMR (75 MHz, $CDCl_3/CD_3OD$, 4:1, epimer A) δ 148.4(C-9), 146.8 (C-8), 137.3 (C-10a), 130.3 (C-2), 128.4 (C-2), 128.6 (C-6a), 128.4 (C-1), 109.7 (C-7), 103.2 (C-10), 101.6 (O$\underline{CH_2}$O), 88.5 (C-6), 78.7 (C-11), 73.4 (C-3), 58.7 (C-12), 57.1 (C-4a), 56.5 (O$\underline{CH_3}$), 51.1 (C-10b), 27.8 (C-4), ppm. ^{13}C NMR (75 MHz, $CDCl_3/CD_3OD$, 4:1, epimer B) δ 148.1 (C-9), 146.9 (C-8), 136.4 (C-10a), 130.3 (C-2), 129.7 (C-6a), 128.6 (C-1), 108.5 (C-7), 103.9 (C-10), 101.5 (O$\underline{CH_2}$O), 86.1 (C-6), 79.5 (C-11), 73.6 (C-3), 62.6 (C-4a), 56.6 (O$\underline{CH_3}$), 52.9 (C-12), 50.6 (C-10b), 28.2 (C-4) ppm.

Hippeastrine (4)

Amorphous powder. $[\alpha]_D^{25}$ + 152.2 (*c* 0.30, $CHCl_3$); lit. $[\alpha]_D^{25}$ + 160 ($CHCl_3$) [2]. ESI-MS (positive mode) m/z (rel. int) 316 $[M + H]^+$ (100). ^{1}H NMR (300 MHz, $CDCl_3$) δ 7.46 (1H, *s*, H-7), 6.95 (1H, *s*, H-10), 6.07 (2H, *dd*, *J* = 4.8, 1.2 Hz, O$\underline{CH_2}$O), 5.66 (1H, *br s*, H-3), 4.59 (1H, *br s*, H-1), 4.40 (1H, *dp*, *J* = 3.7, 1.9 Hz, H-2), 3.15 (1H, *ddd*, *J* = 10.1, 7.3, 3.3 Hz, H-12α), 2.91 (1H, *dd*, *J* = 9.5, 2.2 Hz, H-10b), 2.64 (1H, *d*, *J* = 9.5 Hz, H-4a), 2.52 (2H, *m*, H-11), 2.26 (*q*, *J* = 9.3 Hz, H-12β), 2.06 (3H, *s*, NCH_3), ppm . ^{13}C NMR (75 MHz, $CDCl_3$) δ 164.8 (C-6), 151.9 (C-9), 148.0 (C-8), 145.1 (C-4), 139.4 (C-10a), 118.6 (C-3), 109.9 (C-7), 108.8 (C-10), 82.3 (C-1), 67.2 (C-4a), 66.9 (C-2), 56.2 (C-12), 43.6 (NCH_3), 39.8 (C-10b), 27.9 (C-12), ppm.

Lycorine (5)

Amorphous powder. $[\alpha]_D^{25} - 70.6$ (*c* 0.31, MeOH); lit. $[\alpha]_D^{25} - 83.8$ (EtOH) [2]. IR ν_{max} cm^{-1} (KBr): 3334, 1485, 744. ESI-MS (positive mode) m/z (rel. int) 288 $[M + H]^+$ (100). ^{1}H NMR (300 MHz, DMSO-d_6) δ 6.80 (1H, *s*, H-10), 6.67 (1H, *s*, H-7), 5.95 (2H, *dd*, *J* = 4.1, 0.9 Hz,O$\underline{CH_2}$O), 5.37 (1H, *bs*, H-3), 4.85 (1H, *d*, *J* = 6.2 Hz, 2-OH), 4.75 (1H, *d*, *J* = 4.2 Hz, 1-OH), 4.27 (1H *bs*, H-1), 4.04 (1H, *d*, *J* = 14.2 Hz, H-6β), 3.96 (1H, *bs*, H-2), 3.29 (1H, *d*, *J* = 14.2 Hz, H-6α), 3.18 (1H, *ddd*, *J* = 9.1, 7.2, 2.1 Hz, H12-β), 2.61 (1H, *d*, *J* = 10.4 Hz, H-10b), 2.51 (1H, *m*, H-4a), 2.47 (2H, *m*, H-11), 2.20 (1H, *q*, *J* = 8.5 Hz, H-12α) ppm. ^{13}C NMR (75 MHz, DMSO- d_6) δ 145.6 (C-9), 145.1(C-8), 141.6 (C-4), 129.7(C-6a), 129.5(C-10a), 118.4 (C-3), 106.9 (C-7), 105.0 (C-10), 100.5 (O$\underline{CH_2}$O), 71.7 (C-2), 70.2 (C-1), 60.7 (C-4a), 56.7 (C-6), 53.2 (C-12), 40.1(C-10b), 28.1 (C-11) ppm.

11α-hydroxygalanthamine (6)

Amorphous powder. $[\alpha]_D^{25}$ − 290.3 (*c* 0.13, $CHCl_3$); lit. $[\alpha]_D^{25}$ − 320 [3]. ESI-MS (positive mode) m/z (rel. int) 304 $[M + H]^+$ (100). ^1H NMR (300 MHz, $CDCl_3$) δ 6.66 (1H, *d*, *J* = 8.2 Hz, H-8), 6.59 (1H, *d*, *J* = 8.2 Hz, H-7), 6.06 (1H, *dd*, *J* = 10.2, 5.1 Hz, H-4), 5.77 (1H, *d*, *J* = 10.2 Hz, H-4a), 5.33 (1H, *bs*, H-1), 4.11 (1H, *bt*, *J* = 4.4 Hz, H-3), 3.82 (3H, *s*, OCH_3), 3.78 (1H, *d*, *J* = 14.7 Hz, H-6β), 3.61 (1H, *d*, *J* = 14.7 Hz, H-6α), 3.49 (1H, *m*, H-11β), 3.11 (1H, *bs*, H-12), 2.60 (1H, *bt*, *J* = 15.9, 1.8 Hz, H-2α), 2.57 (3H, *s*, NCH_3), 2.04 (1H, *ddd*, *J* = 15.9, 5.0, 2.5 Hz, H-2β) ppm. ^{13}C NMR (75 MHz, $CDCl_3$) δ 147.1 (C-10), 144.3 (C-9), 130.1 (C-10b), 129.5 (C-4), 127.8 (C-6a), 125.4 (C-4a), 121.3 (C-7), 111.5 (C-8), 83.5 (C-1), 67.2 (C-11), 62.9 (C-6), 61.8 (C-3), 61.0 (C-12), 56.0 (OCH_3), 53.9 (C-10a), 49.2 (N$\underline{CH_3}$), 29.6 (C-2) ppm.

2α-10bα-dihydroxy-9-*O*-demethylhomolycorine (7)

Amorphous powder. $[\alpha]_D^{25}$+ 28.2 (*c* 0.23, MeOH); lit. $[\alpha]_D^{20}$ + 40.2 (MeOH) [4]. ESI-MS (positive mode) m/z (rel. int) 334 $[M + H]^+$ (100). ^1H NMR (300 MHz, DMSO-d_6) δ 7.32 (1H, *s*, H-7), 7.18 (1H, *s*, H-10), 5.57 (1H, *bs*, H-3), 4.74 (1H, *s*, OH), 4.36 (1H, *bs*, H-1), 4.09 (1H, *bs*, H-2), 3.83 (3H, *s*, OCH_3), 3.31 (1H, *s*, OH), 3.16 (1H, *s*, OH), 3.06 (1H, *td*, *J* = 8.4, 3.6 Hz, H-12α), 2.63 (1H, *s*, H-4a), 2.38 (2H, *m*, H-11), 2.22 (1H, *q*, *J* = 8.7 Hz, H-12β), 1.83 (3H, *s*, NCH_3) ppm. ^{13}C NMR (75 MHz, DMSO-d_6) δ 163.6 (C-6), 151.7 (C-9), 147.3 (C-8), 142.0 (C-10a), 140.6 (C-4), 119.1 (C-3), 113.1 (C-6a), 112.2 (C-10), 111.8 (C-7), 83.4 (C-1), 69.6 (C-4a), 67.4 (C-10b), 67.3 (C-2), 55.6 (OCH_3), 55.5 (C-12), 43.5 (NCH_3), 27.9 (C-11) ppm.

Epi-galanthamine (8)

Amorphous powder. $[\alpha]_D^{25}$ − 326.5. (*c* 0.10, MeOH). $[\alpha]_D^{20}$ − 327. (EtOH) [5]. ESI-MS (positive mode) m/z (rel. int) 288 $[M + H]^+$ (100).^1H NMR (300 MHz, $CDCl_3$) δ 6.66 (1H, *d*, *J* = 8.2 Hz, H-8), 6.61 (1H, *d*, *J* = 8.2 Hz, H-7), 6.05 (1H, *dd*, *J* = 10.3, 1.4 Hz, H-4), 6.01 (1H, *ddd*, *J* = 10.3, 4.6, 1.2 Hz, H-4a), 4.60 (1H, *bs*, H-1), 4.13* (1H, *m*, H-3), 4.12* (1H, *d*, *J* = 15.4 Hz, H-6β), 3.82 (3H, s, O$\underline{CH_3}$), 3.69 (1H, *dd*, *J* = 15.2, 1.2 Hz, H-6α), 3.28 (1H, *ddd*, *J* = 14.6, 12.7, 1.9 Hz, H-12β), 3.07 (1H, *dt*, *J* = 14.1, 3.0 Hz, H-12α), 2.68 (1H, *m*, H-2β), 2.40 (3H, *s*, N$\underline{CH_3}$), 2.08 (1H, *ddd*, *J* = 13.6, 10.5, 3.2 Hz, H-11α), 1.99 (1H, *ddd*, *J* = 10.6, 8.3, 2.4 Hz, H-2α), 1.59 (1H, *ddd*, *J* = 13.8, 4.1, 1.9 Hz, H-11β), ppm *overlapped signals. ^{13}C NMR (75 MHz, $CDCl_3$) δ 146.0 (C-9), 144.3 (C-10), 133.1 (C-10a), 128.8 (C-6a), 127.8 (C-4), 126.8 (C-4a), 122.3 (C-7), 111.4 (C-8), 88.8 (C-1), 62.1 (C-3), 60.5 (C-6), 56.0 (O$\underline{CH_3}$), 53.8 (C-12), 48.3(C-10b), 41.8 (NCH_3), 33.7 (C-11), 30.0 (C-2) ppm.

8-*O*-Demethylhomolycorine (9)

Amorphous powder. $[\alpha]_D^{25}$ + 90.7 (*c* 0.092, $CHCl_3$); lit. $[\alpha]_D^{24}$ + 89.6 ($CHCl_3$) [5]. ESI-MS (positive mode) m/z (rel. int) 302 $[M + H]^+$ (100). ^1H NMR (300 MHz, $CDCl_3$) δ 7.59 (1H, *s*, H-7), 7.01 (1H, *s*, H-10), 5.52 (1H, *m*, H-3), 4.76 (1H, *dt*, *J* = 4.4, 2.2 H-1), 3.95 (3H, *s*, OCH_3), 3.22 (1H, *ddd*, *J* = 10.0, 6.1, 4.2 Hz, H-12α), 2.80 (1H, *d*, *J* = 10.0 Hz, H-4a), 2.75 (1H, *dd*, *J* = 9.8, 2.0 Hz, H-10b), 2.61 (1H, *dt*, *J* = 5.4, 2.9 Hz, H-2), 2.50 (2H, *m*, H-11), 2.30 (1H, *dd*, *J* = 18.9, 9.4 Hz, H-12β), 2.00 (3H, *s*, N$\underline{CH_3}$), ppm. ^{13}C NMR (75 MHz, $CDCl_3$) δ 165.8 (C-6), 151.4 (C-9), 146.0 (C-8), 140.3(C-4), 136.5(10a), 117.4 (C-6a), 116.3 (C-7), 115.8 (C-3), 110.6 (C-10), 77.6 (C-1), 66.7 (C-4a), 56.4 (C-12), 56.2 (OCH_3) 43.7 (NCH_3), 43.6 (C-10b), 31.3 (C-2), 27.9 (11), ppm.

Tazettine (**10**)

White crystals, m.p: 208-210 ºC, (hexane/EtOAc) (lit. 208 ºC) [6]. $[\alpha]_D^{25}$ + 130.7 (*c* 0.09, $CHCl_3$); $[\alpha]_D^{22}$ + 145 ($CHCl_3$) [6]. IR: ν_{max} cm^{-1} (KBr): 3348, 2725, 1253, 1119. ESI-MS (positive mode) m/z (rel. int) 332 $[M + H]^+$ (100).^1H NMR (300 MHz, $CDCl_3$) δ 6.85 (1H, *s*, H-12), 6.49 (1H, *s*, H-9), 6.13 (1H, *d*, *J* = 10.4, 3 Hz, H-2), 5.89 (2H, *s*, O$\underline{CH_2}$O), 5.61 (1H, *m*, H-1), 4.95 (1H, *d*, *J* = 14.7 Hz, H-8α), 4.63 (1H, *d*, *J* = 14.8 Hz, H-8β), 4.14 (1H, *m*, H-3), 3.46 (3H, *s*, OCH_3), 3.30 (1H, *d*, *J* = 10.6 Hz, H-6α), 2.87 (1H, *bs*, H-5), 2.68 (1H, *d*, *J* = 10.6 Hz, H-6β), 2.40 (3H, *s*, N-$\underline{CH_3}$), 2.22 (1H, *ddd*, *J* = 15, 5, 3 Hz H-4α); 1.62 (1H, *ddd*, *J* = 15, 6, 3 Hz H-4β) ppm. ^{13}C NMR (75 MHz, $CDCl_3$) δ 146.7 (C-11), 146.5(C-10), 130.7(C-2), 128.8(C-1), 128.1(C-12a), 125.6 (C-8a), 109.5(C-12), 104.1(C-9), 102.1 (C-6a), 101.0 (O$\underline{CH_2}$O), 73.0 (C-3), 70.2 (C-5), 65.6 (C-6), 62.1 (C-8), 56.2 (O$\underline{CH_3}$), 50.0 (C-12b), 42.1 (N-$\underline{CH_3}$), 26.8 (C-4) ppm.

Haemanthamine (11)

Amorphous powder. $[\alpha]_D^{25}$ + 43.7 (*c*, 0.5, $CHCl_3$); lit. $[\alpha]_D^{22}$ + 38.6 ($CHCl_3$) [5]. ESI-MS (positive mode) m/z (rel. int) 302 $[M + H]^+$ (100). ^1H NMR (300 MHz, $CDCl_3$) δ 6.82 (s, 1H, H-10), 6.47 (1H, *s*, H-7), 6.41 (1H, *d*, *J* = 10.1 Hz, H-1), 6.35 (1H, *ddd*, *J* = 10.1, 4.6, 0.8 Hz, H-2), 5.89 (2H, *q*, *J* = 1.4 Hz, O$\underline{CH_2}$O), 4.35 (1H, *d*, *J* = 16.8 Hz, H-6β), 4.00 (1H, *dd*, *J* = 6.6, 3.4 Hz, H-11), 3.85 (1H, *td*, *J* = 4.2, 1.9 Hz, H-3), 3.72 (1H, *d*, *J* = 16.8 Hz, H-6α), 3.40 (1H, *m*, H-12 endo), 3.35 (3H, *s*, O$\underline{CH_3}$), 3.31 (1H, *m*, H-12 exo), 2.11(2H, *m*, H-4).^{13}C NMR (75 MHz, $CDCl_3$) δ 146.7 (C-8), 146.4(C-9), 135.1 (C-10a), 132.2 (C-2), 127.1(C-1), 126.1(C-6a), 106.9 (C-7), 103.5 (C-10), 101.0 (O$\underline{CH_2}$O), 79.9 (C-11), 72.7 (C-3), 63.4 (C-12), 62.9 (C-4a), 61.2 (C-6), 56.7 (O$\underline{CH_3}$), 50.3 (C-10b), 28.1(C-4) ppm.

NMR spectra of some compounds

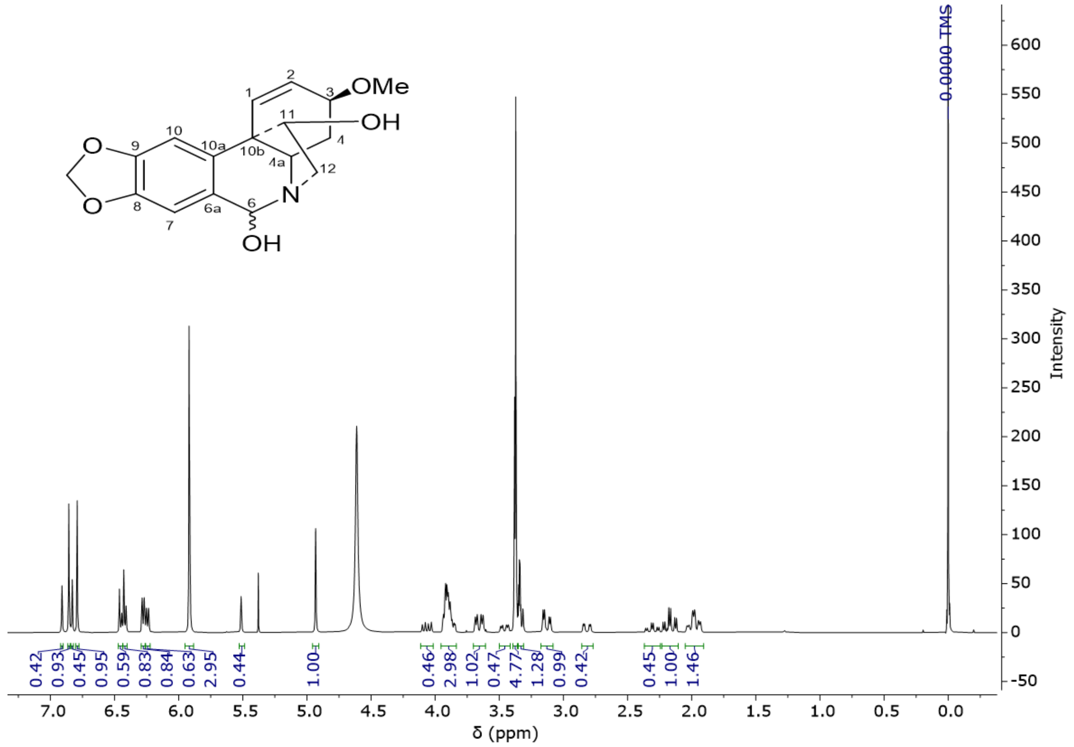

Figure S1. ^{1}H-NMR spectrum of compound **3** (300 MHz, $CDCl_3/CD_3OD$).

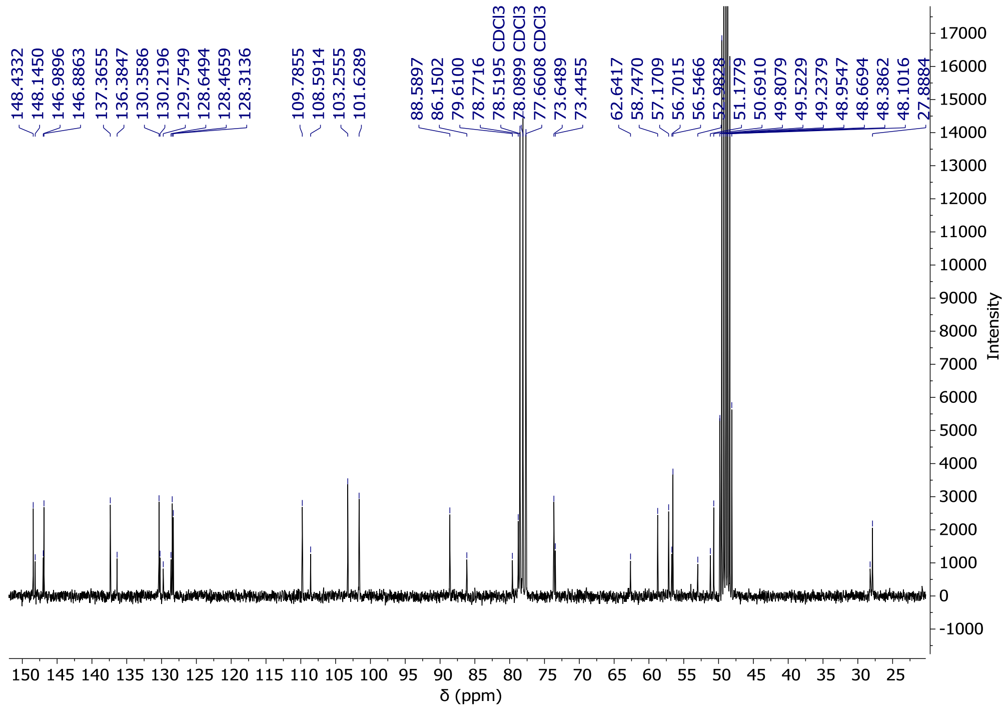

Figure S2. ^{13}C-NMR spectrum of compound **3** (75 MHz, $CDCl_3/CD_3OD$).

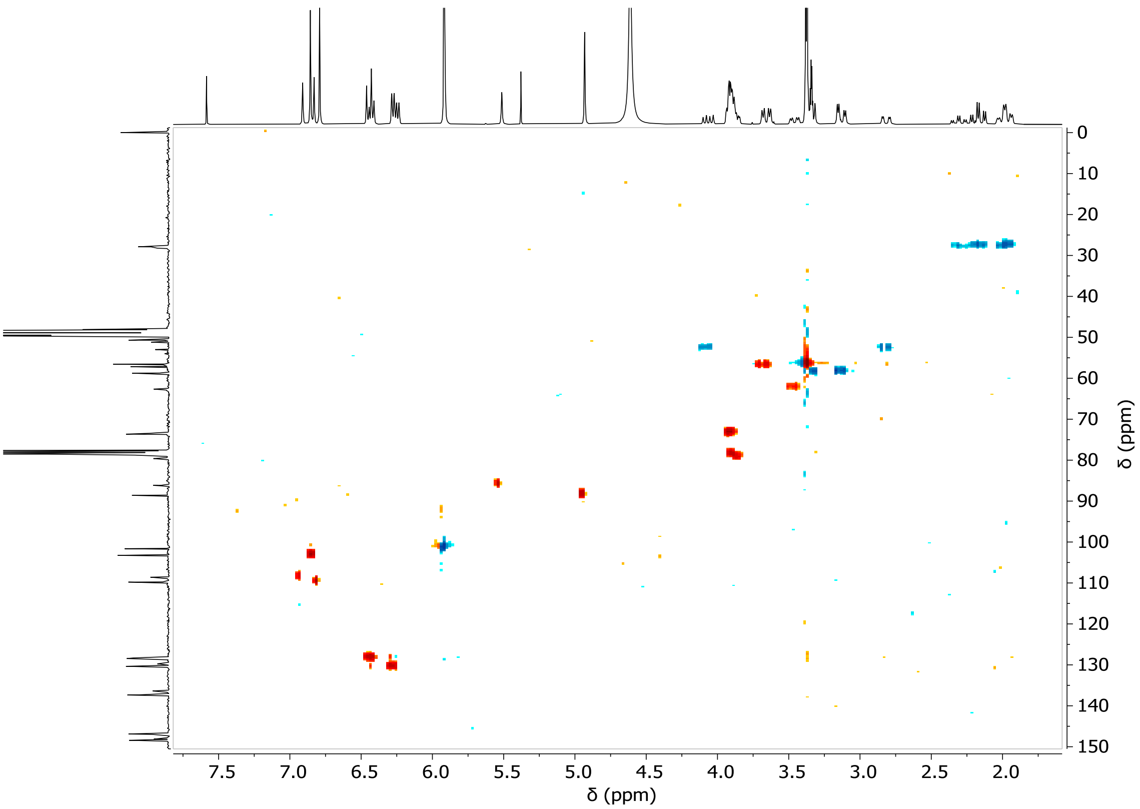

Figure S3. HMQC spectrum of compound **3**.

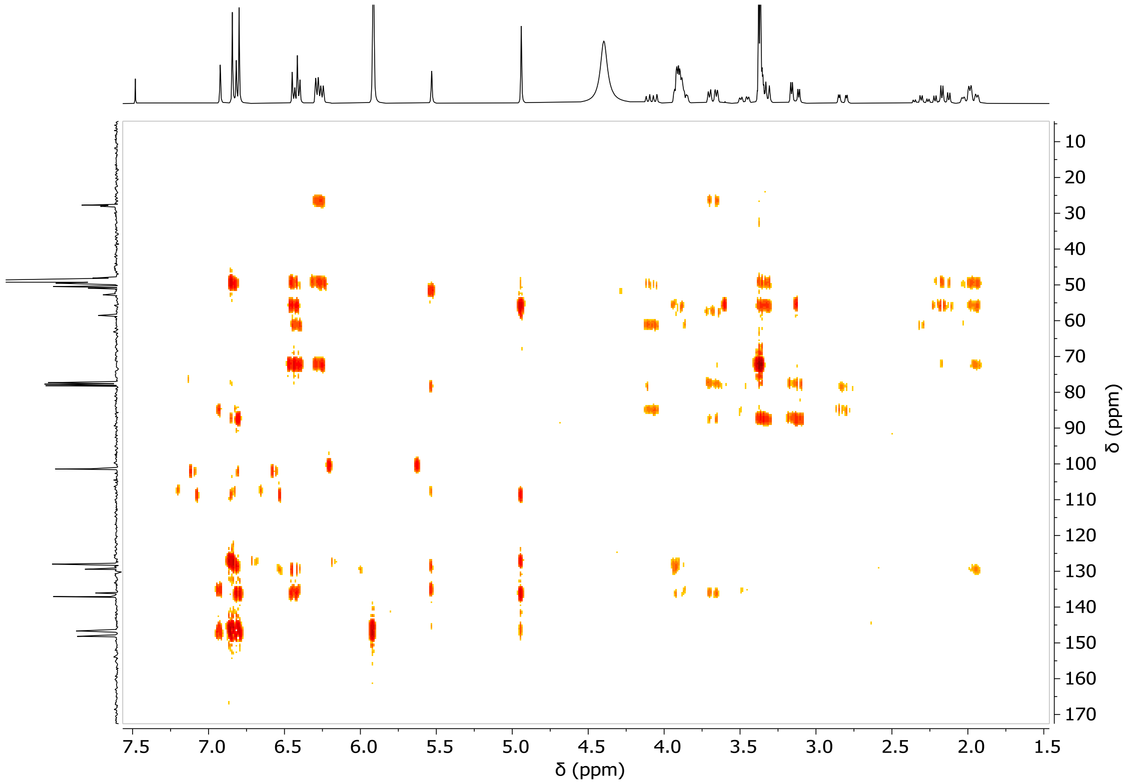

Figure S4. HMBC spectrum of compound **3**.

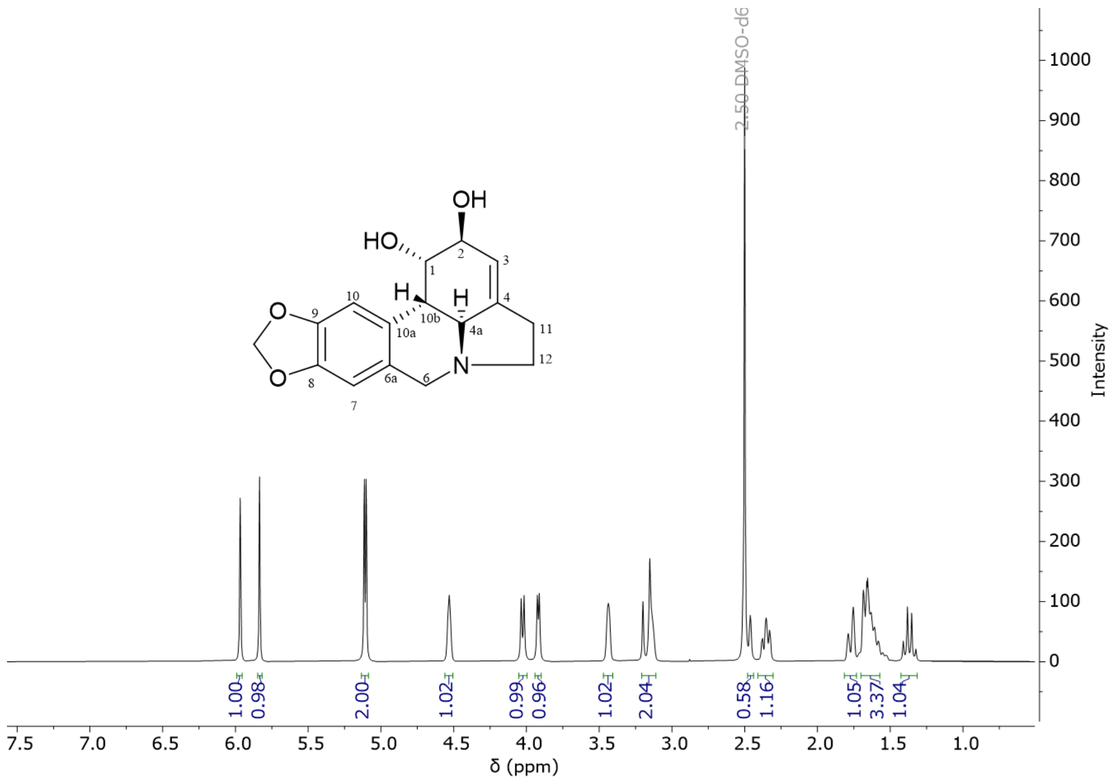

Figure S5. ^{1}H-NMR spectrum of compound **5** (300 MHz, DMSO-*d*6).

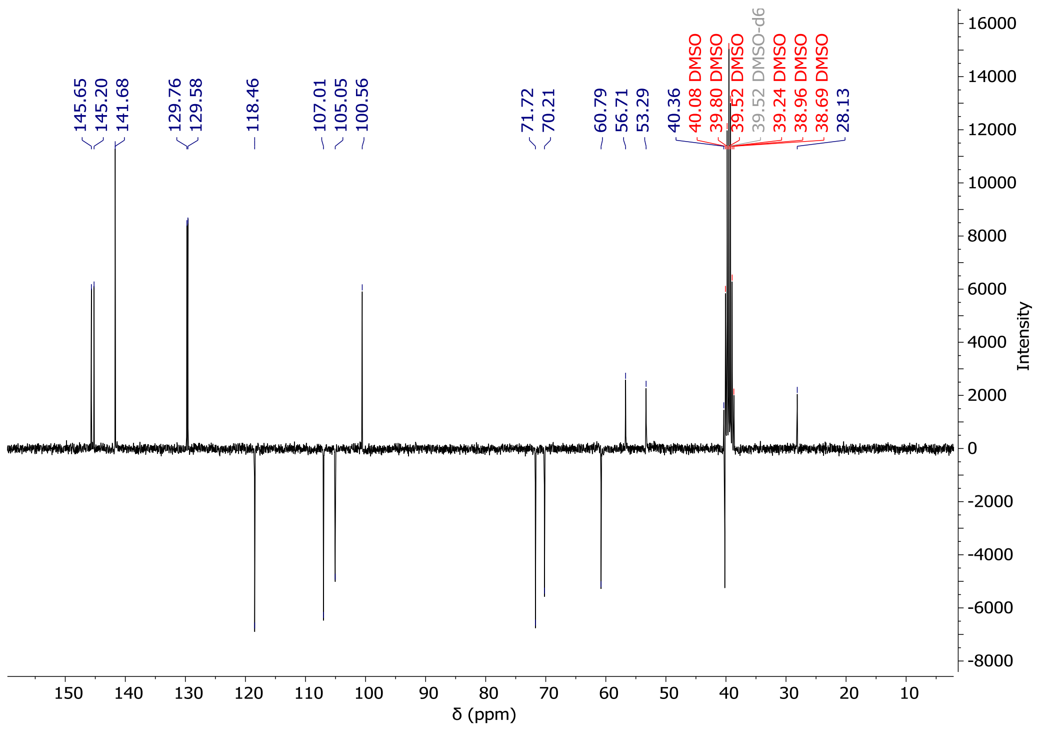

Figure S6. ^{13}C-APT NMR spectrum of compound **5** (75 MHz, DMSO-*d*6).

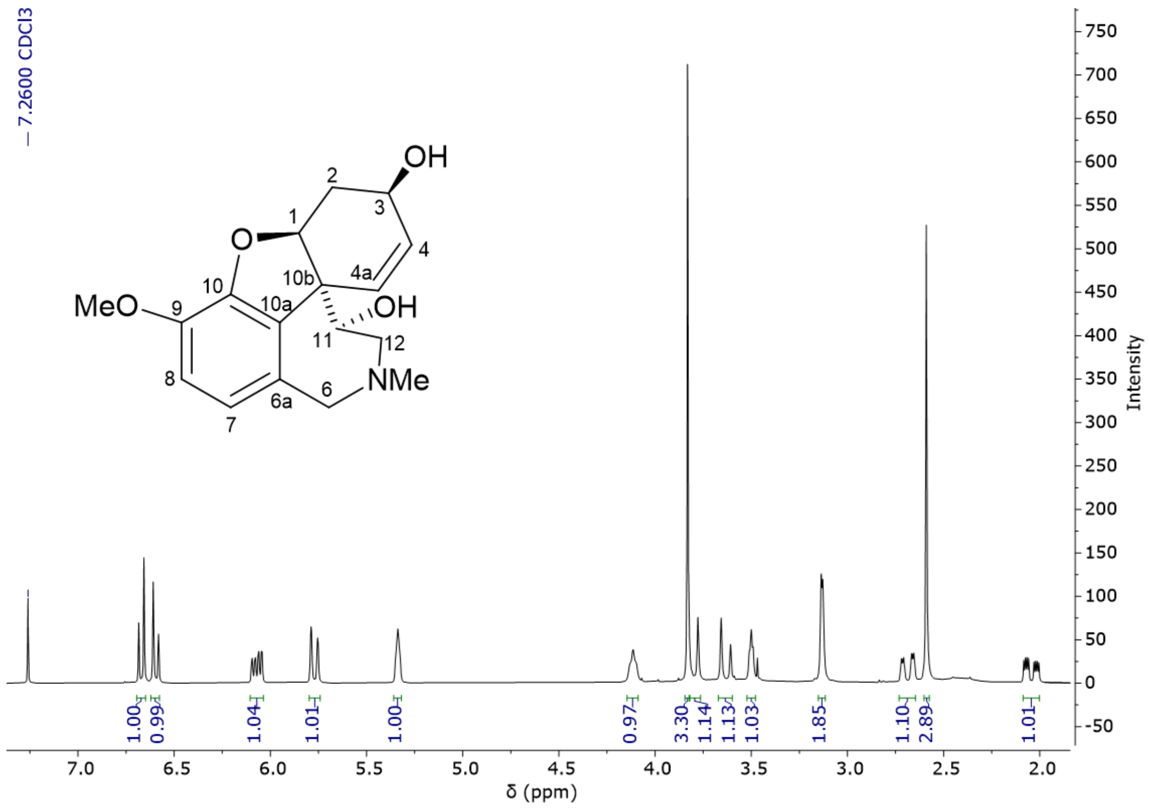

Figure S7. ^{1}H-NMR spectrum of compound **6** (300 MHz, $CDCl_3$).

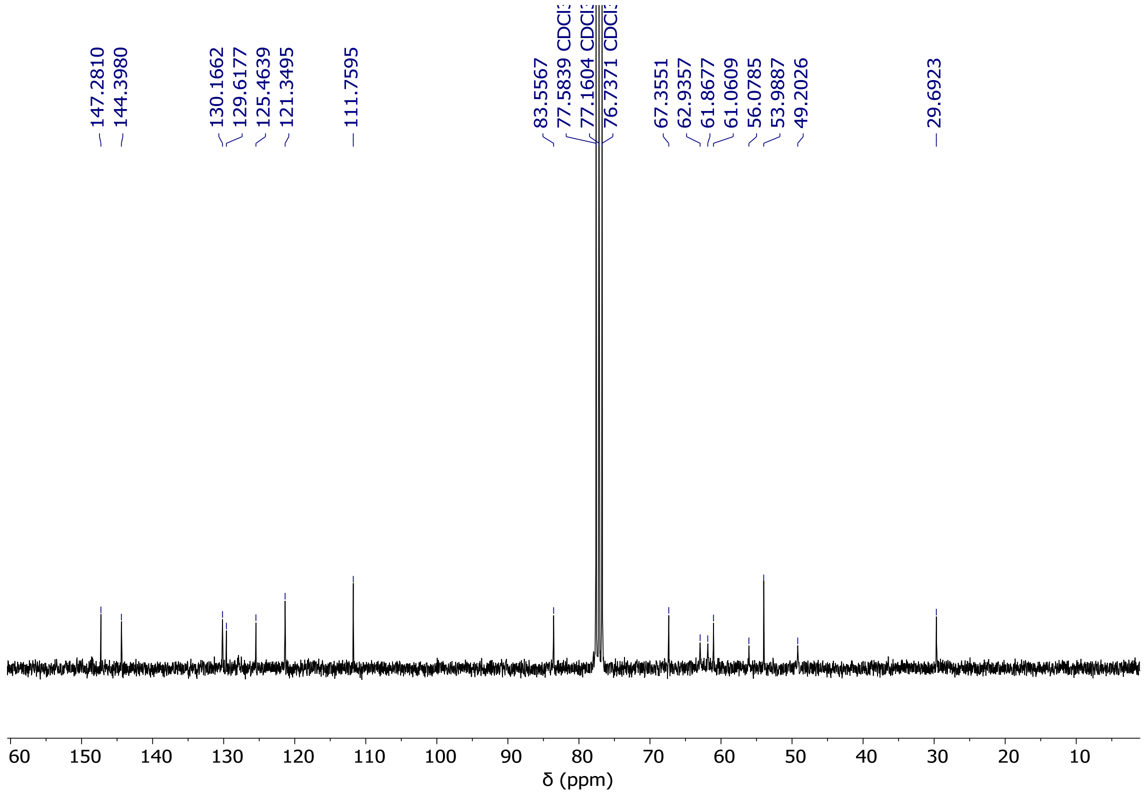

Figure S8. ^{13}C-NMR spectrum of compound **6** (75 MHz, $CDCl_3$).

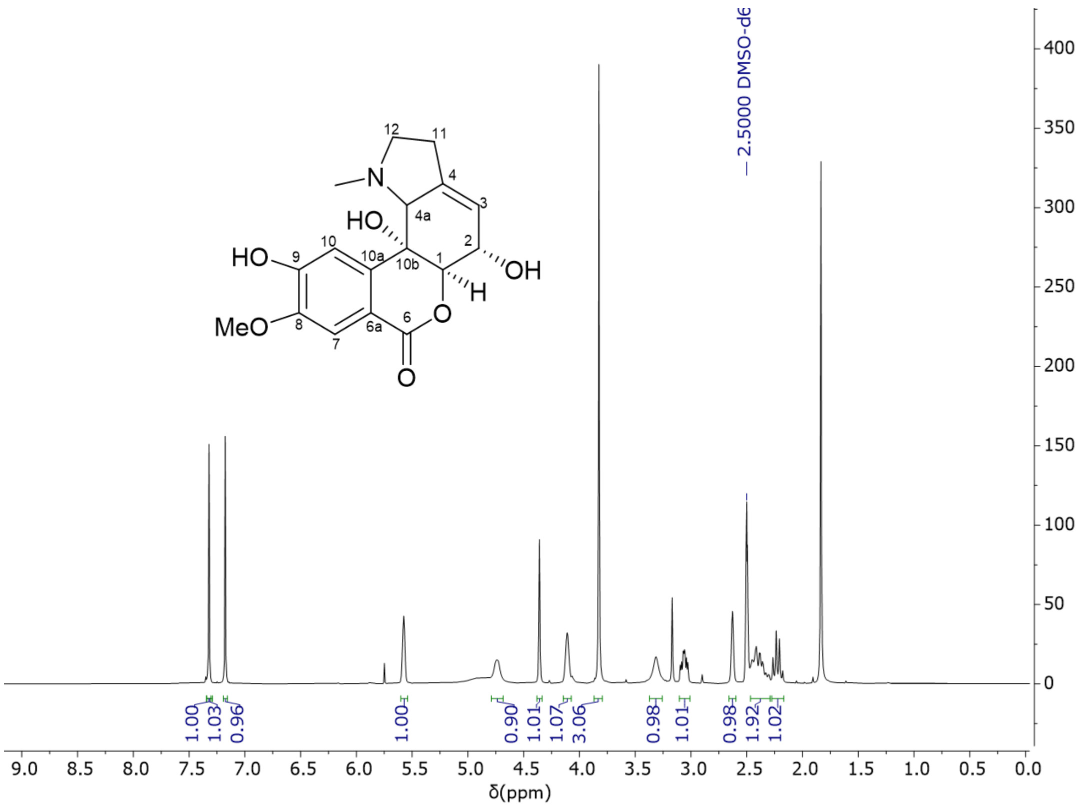

Figure S9. ^{1}H-NMR spectrum of compound **7** (300 MHz, DMSO-*d*$_6$).

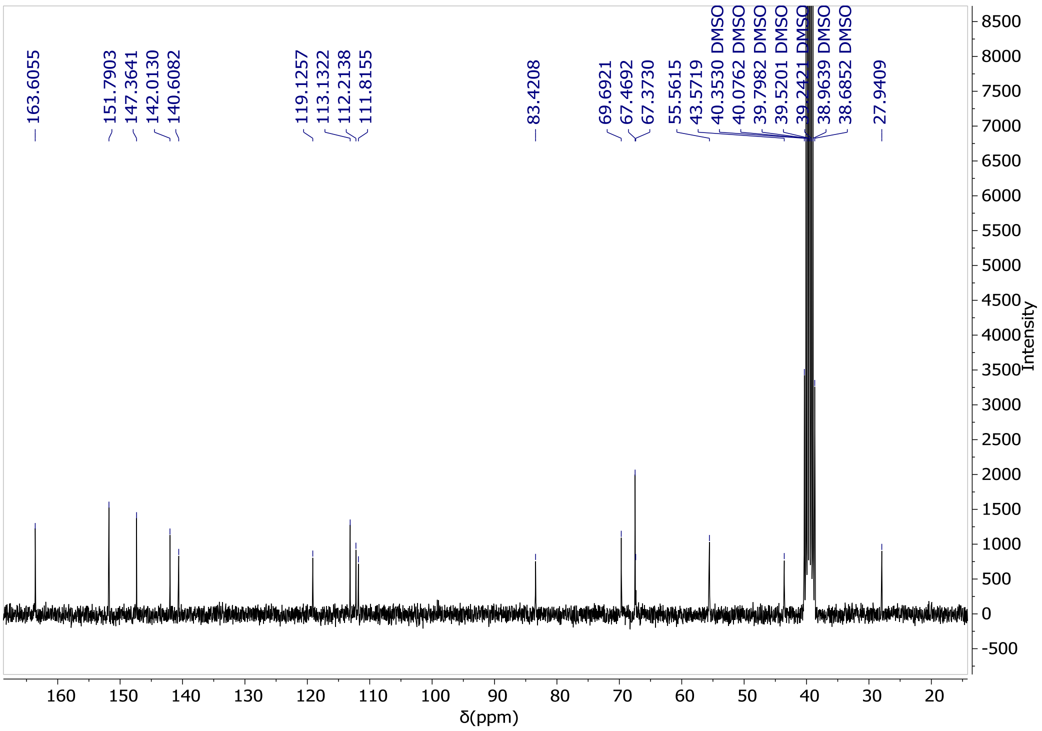

Figure S10. ^{13}C NMR spectrum of compound **7** (75 MHz, DMSO-*d*$_6$).

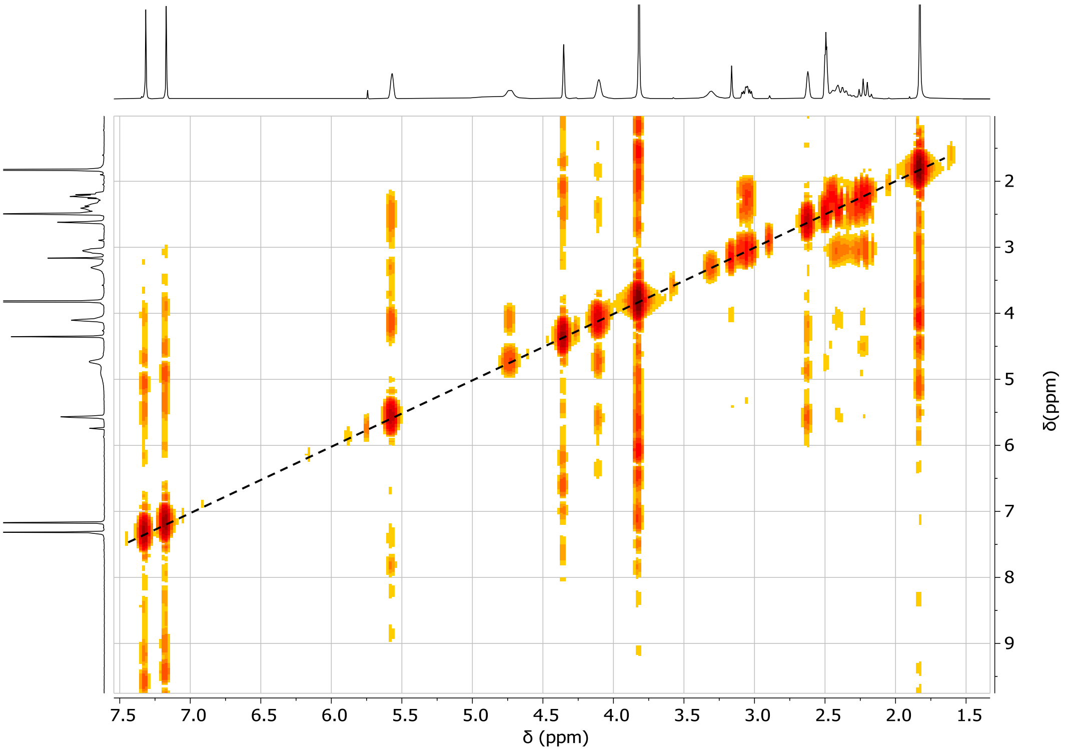

Figure S11. COSY spectrum of compound **7**.

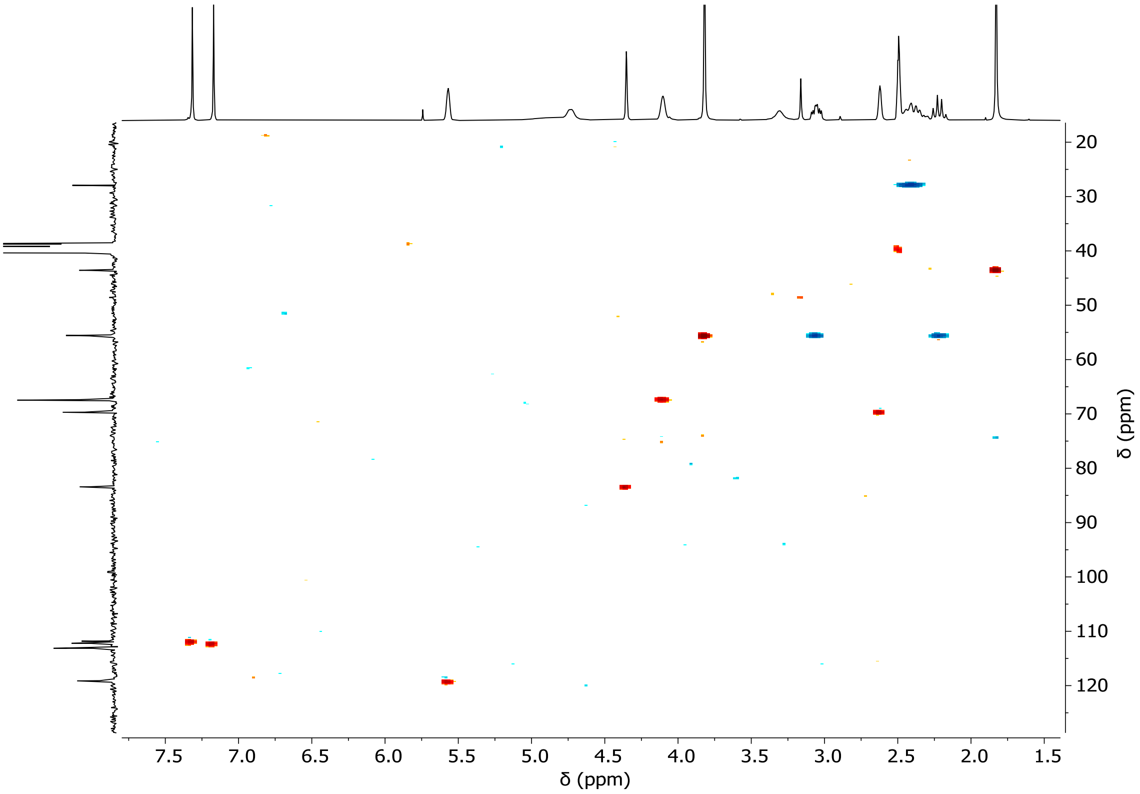

Figure S12. HMQC spectrum of compound **7**.

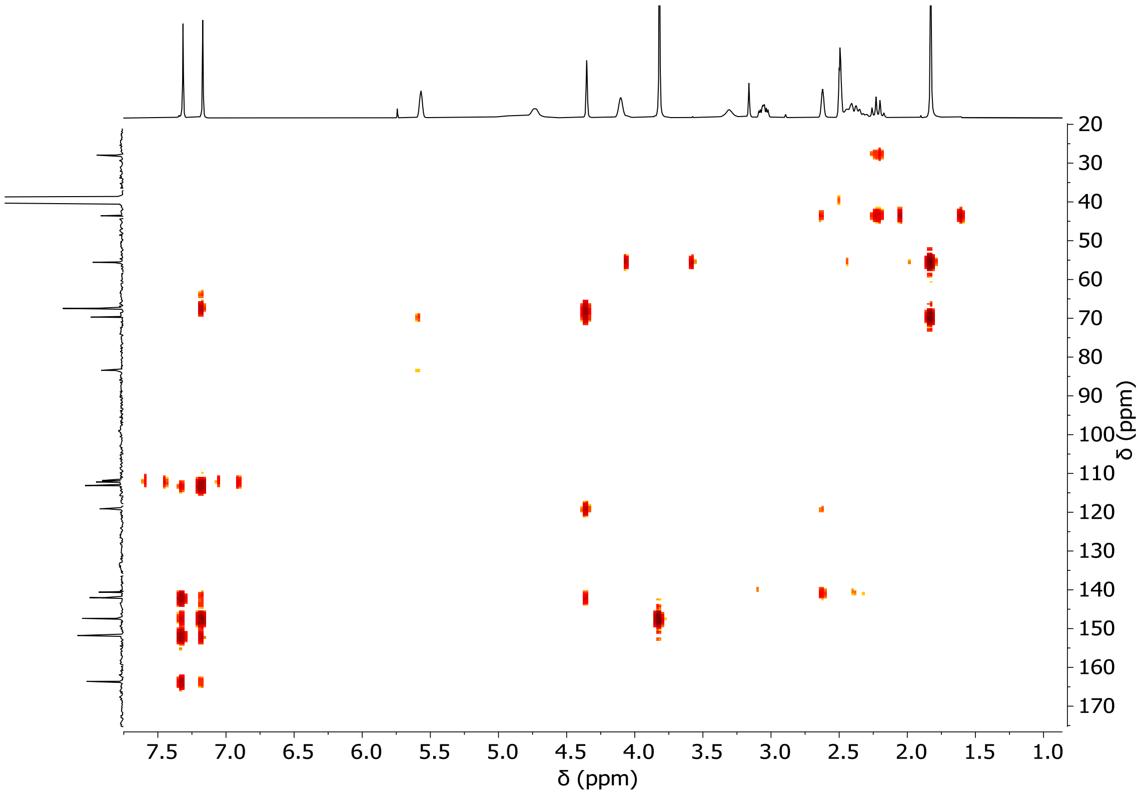

Figure S13. HMBC spectrum of compound **7**.

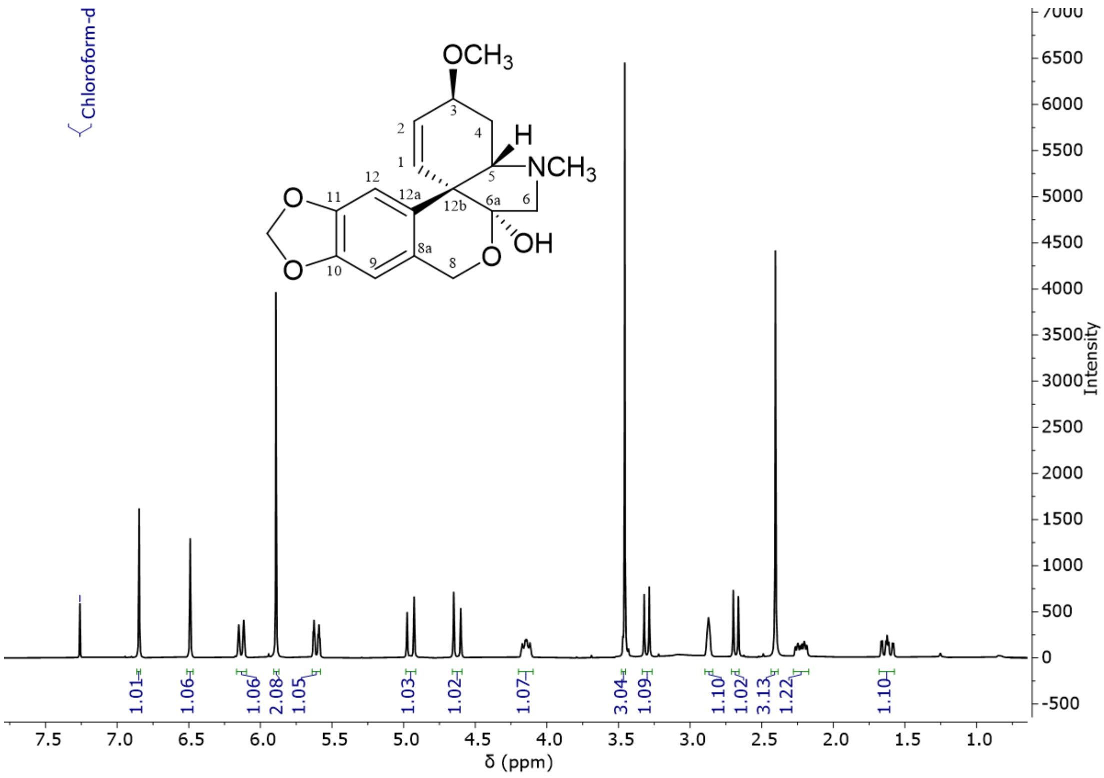

Figure S14. ^{1}H-NMR spectrum of compound **10** (300 MHz, $CDCl_3$).

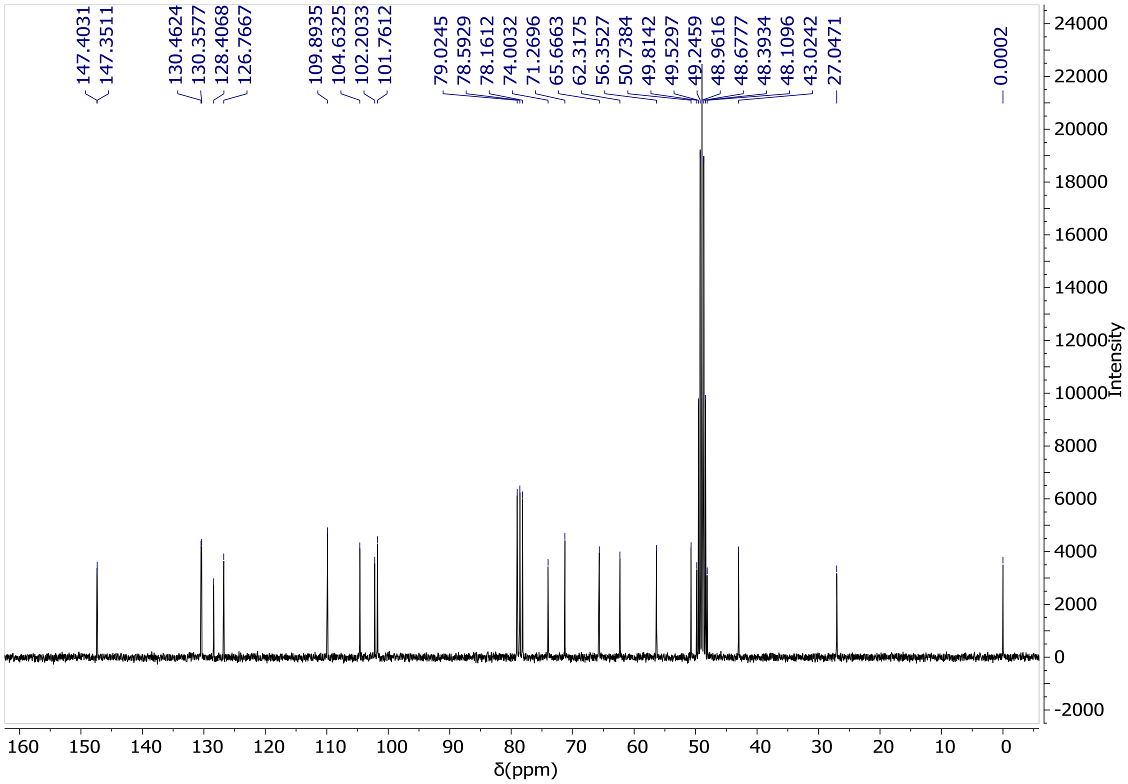

Figure S15. ^{13}C-NMR spectrum of compound **10** (75 MHz, $CDCl_3/CD_3OD$).

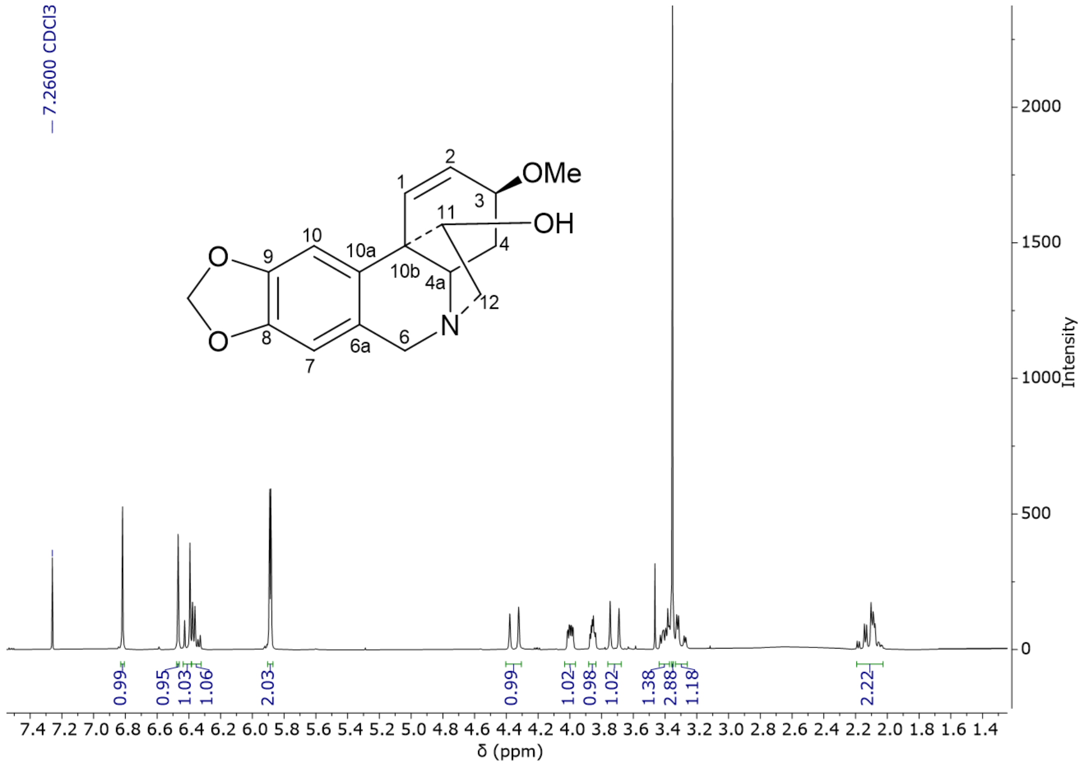

Figure S16. ^{1}H-NMR spectrum of compound **11** (300 MHz, $CDCl_3$).

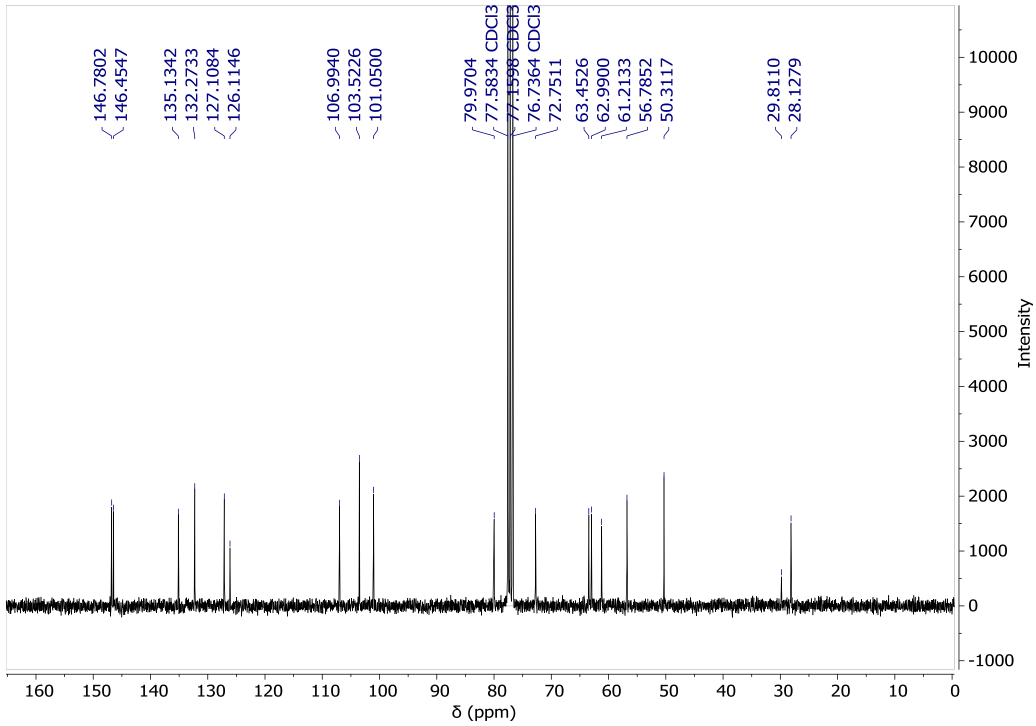

Figure S17. ^{13}C-NMR spectrum of compound **11** (75 MHz, $CDCl_3$).

References

1. Fukuda, N.; Yonemitsu, M.; Kimura, T. Studies on the Constituents of the Stems of Tinospora Tuberculata N-Trans and N-Cis-Feruloyl Tyramine, and a New Phenolic Glucoside Tinotuberide. *Chem. Pharm. Bull* **1983**, *31(1)*, 156–161.

2. Ghosal, S.; S. Saini, K.; Razdan, S. Crinum Alkaloids: Their Chemistry and Biology. *Phytochemistry* **1985**, *24*, 2141–2156.

3. Wildman, W.C.; Brown, C.L. The Structure of Habranthine. *Tetrahedron Lett.* **1968**, *9*, 4573–4576.

4. Carvalho, K.R.; Silva, A.B.; Torres, M.C.M.; Pinto, F.C.L.; Guimarães, L.A.; Rocha, D.D.; Silveira, E.R.; Costa-Lotufo, L. V.; Braz-Filho, R.; Pessoa, O.D.L. Cytotoxic Alkaloids from Hippeastrum Solandriflorum Lindl. *J. Braz. Chem. Soc.* **2015**, *26*, 1976–1980.

5. Bastida, J.; Viladomat, F.; Codina, C. Narcissus Alkaloids. In *Studies in Natural Products Chemistry*; 1997; Vol. 20, pp. 323–405.

6. Döpke, W.; Pham, L.H.; Gründemann, E.; Bartoszek, M.; Flatau, S. Alkaloids from Hip Peastrum Equestre ; Part I : Phamine , a New Phenanthridone Alkaloid. *Planta Med* **1995**, *61*, 564–566.
